# Uric Acid and Hypertension: Prognostic Role and Guide for Treatment

**DOI:** 10.3390/jcm10030448

**Published:** 2021-01-24

**Authors:** Federica Piani, Arrigo F. G. Cicero, Claudio Borghi

**Affiliations:** 1 Department of Medical and Surgical Sciences, University of Bologna, 40138 Bologna, Italy; 2 IRCCS, Azienda Ospedaliero, Universitaria di Bologna, 40138 Bologna, Italy; federicamariasolepiani@gmail.com (F.P.); arrigo.cicero@unibo.it (A.F.G.C.)

**Keywords:** uric acid, hypertension, cardiovascular risk, urate-lowering therapy, xanthine oxidase

## Abstract

The relationship between serum uric acid (SUA) and hypertension has been a subject of increasing interest since the 1870 discovery by Frederick Akbar Mahomed. Several epidemiological studies have shown a strong association between high SUA levels and the presence or the development of hypertension. Genetic analyses have found that xanthine oxidoreductase (XOR) genetic polymorphisms are associated with hypertension. However, genetic studies on urate transporters and Mendelian randomization studies failed to demonstrate a causal relationship between SUA and hypertension. Results from clinical trials on the role of urate-lowering therapy in the management of patients with hypertension are not uniform. Our study sought to analyze the prognostic and therapeutic role of SUA in the hypertensive disease, from uric acid (UA) biology to clinical trials on urate-lowering therapies.

## 1. Introduction

Hypertension is the leading cause of premature death and cardiovascular diseases (CVD) worldwide [1]. The prevalence of CVD and hypertension has not decreased throughout the last decades, and CVD is still responsible for nearly 33% of all global deaths [1,2]. Potential explanations include unrecognized or undertreated risk factors for the development and progression of the hypertensive disease, such as uric acid (UA). The first report of the association between serum UA (SUA) and hypertension dates back to 1879, by Frederick Akbar Mahomed [3]. Since then, numerous studies have confirmed this strong association [4,5,6,7,8,9,10,11,12,13,14,15,16,17,18,19,20]. Several mechanisms have been proposed to explain the potential role of SUA in the development of hypertension, including UA-mediated kidney afferent arteriolopathy, renin-angiotensin-aldosterone system (RAAS) activation, oxidative stress, inflammation, and endothelial dysfunction [21,22,23,24,25,26,27,28,29,30,31,32,33]. However, the precise pathophysiologic patterns remain elusive. Genetic population-based association analyses have shown a correlation between xanthine oxidoreductase (XOR) genetic polymorphisms and hypertension [34,35,36,37]. However, polymorphisms involving major urate transporters genes (e.g., SLC2A9) have not been demonstrated to correlate with hypertension [38,39]. XOR is involved in intracellular UA production, while urate transporters play a major role in regulating extracellular UA concentration and UA excretion [40,41,42,43]. Thus, the results of genetic studies may underlie a critical role of intracellular UA production in the development and progression of hypertension. This could explain the heterogeneous effects of UA-lowering drugs on blood pressure (BP), with some studies showing no effect [44,45,46,47,48,49], and others demonstrating a reduction on BP values [50,51,52,53,54,55,56,57,58,59]. Indeed, the effects of UA-lowering drugs on intracellular UA concentrations have not been fully elucidated and may explain the different response on BP to these therapeutic agents. The aim of this review is to present the available evidence on the relationship between UA and hypertension with an emphasis on the prognostic and therapeutic role of UA and UA-lowering medications on the hypertensive disease.

## 2. Materials and Methods

To synthesize a summary of studies evaluating the relationship between UA and hypertension, we performed a comprehensive literature review. The following key words were used to search the literature “hypertension”, “hypertensive disease”, “hypertensive treatment”, “anti-hypertensive treatment”, “beta-blockers”, “diuretics”, “RAAS inhibitors”, “alpha-1 antagonists”, “calcium channel blockers”, “uric acid”, “hyperuricemia”, “gout”, “urate-lowering therapies”, “allopurinol”, “febuxostat”, “uricosuric agents”, “probenecid”, “pegloticase”, and “rasburicase”.

No search restrictions were imposed, and references were scanned to identify other potentially relevant studies.

## 3. Discussion

### 3.1. UA Biology

UA is a weak acid, and in normal blood pH values it exists predominantly as urate anion. XOR catalyzes the generation of urate from hypoxanthine and xanthine, which are the last metabolites of endogenous and exogenous purine mononucleotide catabolism [40,42,60]. XOR has two forms: Xanthine dehydrogenase (XDH), the most prevalent, and xanthine oxidase (XO) [61]. Hyperuricemia is defined as SUA > 6 mg/dL in women, SUA > 7 mg/dL in men, and SUA > 5.5 mg/dL in the pediatric population, and its prevalence is increasing worldwide [42,62]. During the Miocene epoch, humans lost the capacity to metabolize UA into allantoin due to nonsense mutations in the gene codifying the enzyme uricase [63]. However, a minimal amount of UA can be indirectly metabolized through its reaction with oxidants, lipid radicals, nitric oxide, and peroxynitrite [41].

The most common cause of hyperuricemia is a decrease in UA excretion, e.g., during renal insufficiency, metabolic acidosis, hypothyroidism, or treatment with drugs such as beta blockers [42]. Increased purine degradation (e.g., during DNA, RNA, and ATP breakdown) also leads to a rise in SUA [40]. In addition, increased activity of aldose reductase and XO, as occurring during ischemia, heat stress, and dehydration, have been associated with a rise in intracellular UA and SUA.

Approximately two-thirds of urate elimination occurs in the kidney, while one-third occurs in the small intestine. In the kidney, UA is readily filtered, and up to 90% is reabsorbed by the proximal tubular cells by the apical transporters URAT1 and OAT4, and the basolateral GLUT9 [42]. In addition, UA can be secreted in variable amounts into the proximal tubular lumen by the apical transporters ABCG2, NPT1 and 4, SLC2A9b, and the basolateral OAT1 and 3 [42,43]. The intestinal elimination of UA is mediated by both bacterial uricolysis (mainly for orally administered purine-high aliments) and cellular excretion by the transporters P-Glycoprotein, MCT9, NPT4, NPT homolog (NPT5), OAT10, GLUT9, ABCG2, and MRP2 and 4 [64]. Recently, other UA transporters have been discovered with genome wide-association studies, increasing the UA-homeostatic system knowledge complexity [43,64].

The so-called UA remote sensing and signaling theory has hypothesized an interplay between gut microbiome, kidney urate transporters, and gut urate transporters in the regulation of SUA levels [43]. Interestingly, the composition of the gut microbiota has shown to play a role in UA metabolism in both human and animal models [65,66,67]. For example, higher prevalence of Escherichia coli has been associated with a greater extent of intestinal UA decomposition [65]. The transplant of fecal microbiota of hyperuricemic rats to healthy rats was able to induce an increase in SUA concentrations [66]. Additionally, in rat models, bariatric surgery has been shown to alter the composition of the gut microbiota, with a resulting reduction in SUA concentration [67].

### 3.2. Biology of the Association between UA and Hypertension

UA has demonstrated a crucial role in the pathogenesis of hypertension and kidney disease progression [41,42,68]. Possible pathophysiological mechanisms involve RAAS upregulation, kidney afferent arteriolopathy, endothelial dysfunction, oxidative stress, and systemic inflammation.

Experimental studies on human cell cultures and hyperuricemic animal models have shown that UA can upregulate the renin-angiotensin-aldosterone system (RAAS) [21,22,23,24,25,26]. In addition, RAAS can be indirectly activated by UA-mediated inflammatory status [23,69]. Some in vivo human studies showed a correlation between SUA and RAAS [70,71], while others did not [72,73]. A possible explanation for these inconsistent results is that most studies analyzed systemic RAAS activity and not specifically intrarenal RAAS. In fact, intrarenal RAAS could be upregulated by UA without a concomitant increase in systemic RAAS activity. Supporting this hypothesis, a study on 249 adults has shown that SUA correlated with intrarenal RAAS activity, but not with plasma renin activity [74].

Other than triggering RAAS, UA has been shown to induce hypertension through a crystal and pressure-independent kidney afferent arteriolopathy [27,28,29]. Afferent arteriolopathy can indeed lead to impaired renal blood flow, ischemia, and consequent renovascular hypertension [75,76]. However, the exact mechanism whereby UA can induce arteriolopathy remains partially unsolved. UA-induced oxidative stress and inflammation may play a role, being responsible for the pathogenesis of endothelial dysfunction [24,25,26,32,33]. Another possible cause of afferent arteriolopathy is the UA-mediated vascular smooth muscle cell proliferation [25,31,77,78]. UA-mediated hypertension may also be caused by the up-regulation of thromboxane and endothelin-1. In fact, both these vasoconstrictor molecules can be over-expressed in the presence of high UA concentrations, inducing the development of hypertension [65,79]. UA can also mediate the development of hypertension through a crystal-dependent pathway [80,81]. In fact, monosodium urate deposits have been found in the kidney medulla, cardiac valves, arteries, and within the atherosclerotic plaque [80,81]. These deposits could lead to kidney injury and arterial stiffness, increasing the risk for hypertension [80,81].

Biological variables that could influence the association between UA and hypertension are sex and women’s menopausal state [82,83]. Indeed, it has been demonstrated that postmenopausal women had a stronger association between high SUA and hypertension, compared to premenopausal women [82]. Additionally, Nishio and colleagues have shown that women had a stronger association between high SUA and hypertension compared to men, after multivariate adjustment [83]. Further studies are warranted to fully elucidate the role of sex and menopausal state in the association between SUA and hypertension.

### 3.3. Genetic Studies on the Association between UA and Hypertension

In the last decades, an increasing number of genome wide association studies and Mendelian randomization studies have attempted to validate the epidemiological association observed between SUA and hypertension [84,85]. Genome wide association studies in European, African American, and East Asian populations have shown that genes encoding urate transporters (e.g., GLUT9, URAT1, ABCG2) were the main cause for SUA levels, and that the heritability of SUA in Europeans was estimated ~27–41% [84]. Different studies demonstrated that polymorphisms of the SLC2A9 gene associate with the risk of hypertension [85]. Additionally, aldehyde dehydrogenase II (ALDH-2) polymorphisms have been associated with SUA levels and hypertension in genome wide association studies [86]. Other genetic polymorphisms that regulate SUA levels and associate with hypertension are XOR gene variants [85,87,88]. Of note, XOR is not only associated with increased SUA levels, but also with increased oxidative stress, which in turn may lead to the development of hypertension [87,88]. In contrast with the aforementioned studies, the genetic risk score developed using 30 gene variants responsible for SUA levels has been associated with lower BP values [84]. Additionally, Mendelian randomization studies failed to demonstrate a causal relationship between SUA and the development of hypertension [41,84].

Possible explanations for these contrasting results include the role of intracellular UA, the use of diuretics, and the dietary sodium intake [41]. Intracellular UA concentrations are not always associated with SUA levels and are responsible for the biological effects of UA [41]. It is still unknown how urate transporters regulate intracellular UA levels. Indeed, different polymorphisms of SCL2A9 gene have been shown different effects ranging from hyperuricemia without hypertension (liver specific knockout) to hyperuricemia and hypertension (intestinal knockout) or hypouricemia (systemic knockout) [41]. In animal models, high salt diet has shown to induce intracellular hepatic urate generation and to increase BP values without a parallel increase in SUA concentration [89]. In addition, randomized controlled clinical trials have shown that high dietary sodium intake (200–250 mmol/day) was associated with lower SUA levels and higher BP values [90].

Finally, as suggested by Kei and colleagues, the association of the genetic risk score for hyperuricemia with lower BP values might be explained by the possible confounding effect of the treatment with diuretics [85]. In fact, people with a high risk score have a higher probability to develop hypertension and thus to be under diuretic treatment. In addition, treatment with diuretics is known to induce an increase in SUA concentrations [41,85].

### 3.4. Prognostic Role of UA in Hypertension

One of the main challenges in determining the association between UA and hypertension has been the coexistence of hyperuricemia with other cardiovascular (CV) risk factors. For example, SUA levels are usually increased in people with metabolic syndrome, which is a known risk factor for hypertension [91]. To determine if UA was an independent risk factor for hypertension, most epidemiologic studies have used multivariate analyses and adjustments.

Several cross-sectional studies have shown that hyperuricemia is present in 25–60% of individuals with untreated essential hypertension, and SUA levels are associated with prehypertension [92,93,94]. Longitudinal studies have confirmed the prognostic value of UA in hypertension, demonstrating that higher SUA levels were associated with an increased relative risk for hypertension [4,5,6,7,8,9,10,11,12,13,14,15,16,17,18,19,20] (Table 1).

Large meta-analyses confirmed the same findings, however Mendelian Randomization studies failed to demonstrate a causal relationship between SUA and hypertension [95,96,97]. Interestingly, genetic population-based association analyses have shown that XOR genetic polymorphisms, but not major urate transporters ones, associated with hypertension [34,35,36,37,38,39]. Since XOR is involved in intracellular UA production, the results of those genetic studies may underlie a critical role of intracellular UA production, instead of SUA, in the development and progression of hypertension. Supporting this hypothesis, plasma XO activity has been associated with CV outcomes, independently of SUA levels [98,99]. In addition, Boban et al. showed that XOR was upregulated in subjects with essential hypertension [100].

### 3.5. UA Lowering-Therapies Effects on Blood Pressure

There are two main classes of hypouricemic agents: UA production inhibitors and UA excretion promoters [101,102]. So far, there have been only a few studies that have analyzed the effects of UA-lowering drugs in patients with hypertension [44,45,46,48,49,50,51,52,53,54,55,56,57,58,59,103] (Table 2). Allopurinol, febuxostat, probenecid, and pegloticase all demonstrated antihypertensive properties [50,51,52,53,54,55,56,57,58,59].

Children and adolescents with hyperuricemia and prehypertension or hypertension showed a greater decrease in BP values after allopurinol or probenecid initiation, compared to placebo [50,51,55]. In another controlled study, allopurinol treatment was able to decrease systolic BP values in adults with overweight and prehypertension [52]. Interestingly, allopurinol has also shown a long-term effect in decreasing central blood pressure in adults with recent ischemic stroke or transient ischemic attack [56]. UA lowering-drugs have demonstrated an antihypertensive effect in populations with hypertension and hyperuricemia [50,54,59]. Indeed, the population of the studies that showed no BP effects of hypouricemic drugs did not include subjects with both hypertension and hyperuricemia [44,45,46,48,49,103].

Most studies on subjects with chronic kidney disease (CKD) did not show any antihypertensive property of UA-lowering medications [44,45,49]. A possible explanation is that several factors other than UA drive BP in subjects with CKD. Furthermore, since people with CKD are commonly prescribed with RAAS inhibitors and RAAS activation could be one of the UA-mediated hypertension mechanisms, in this population, the antihypertensive effect of UA-lowering drugs could be masked [21,22,23,24,25,26,66].

Overall, the results of the available studies appear controversial. Possible explanations include the different study designs, the small and heterogeneous populations, the presence of confounding factors, the complex relationship between SUA and intracellular UA, and the short follow-up periods.

Several studies have shown a U-shaped association between SUA concentrations and different CV outcomes [104]. Additionally, a U-shaped association between SUA and microvascular remodeling indexes has been recently demonstrated [105]. The U-shaped relationship between SUA and different CV indexes may explain the heterogeneity of UA-lowering medications trials results.

Large clinical trials on the BP effects of UA-lowering drugs in specific study populations are warranted to further clarify the benefits of this class of drugs in the treatment of hypertension and its complications.

### 3.6. Antihypertensive Medications Effects on SUA

Most antihypertensive medications, such as RAAS inhibitors, alpha-1 blockers, beta blockers, and diuretics, have been shown to increase SUA levels, with an enhanced risk of incident gout [106,107].

On the other side, calcium channel blockers and the angiotensin II receptor blocker losartan have been shown to reduce SUA concentrations and the risk of gout [108,109,110]. In particular, losartan has been shown to reduce SUA levels when administered alone [106] or, to a greater extent, in combination with the calcium channel blocker amlodipine [108]. Interestingly, in The Losartan Intervention. For Endpoint reduction in hypertension (LIFE) study, the increased CV risk reduction obtained with losartan compared to atenolol was mainly attributable to the UA-lowering properties of losartan [104]. The mechanism of losartan reduction of SUA seems to involve the inhibition of UA reabsorption with a uricosuric effect [110].

Altogether these findings underlie the importance of the so-called personalized medicine. In other words, it appears mandatory to take into account the characteristics of the single patient in order to choose the appropriate antihypertensive drugs. This approach also leads to a holistic view of CV risk, with a potential improvement of preventive and therapeutic strategies to reduce CVD burden.

### 3.7. How to Choose the Right Hypouricemic Agent

A recent systematic review of clinical practice guidelines and consensus statements on the treatment of hyperuricemia and gout recommended long-term treatment of patients with gout or with comorbities and SUA > 6.0 mg/dL (or 360 μmol/L) [102]. Even if treatment of asymptomatic patients without comorbities is still not recommended, large population studies such as the multicenter Uric Acid Right for Heart Health (URRAH) Study showed that the optimal SUA cut-off for reducing CV mortality was under 5.6 mg/dL [111,112,113].

The recommended first-line treatment was allopurinol, unless the patient had the genetic variant HLA-B*5801, which is commonly found in Han Chinese, South East Asians, and African Americans [102].

The second line treatment is febuxostat [102]. If the target SUA level is not reached, a combination therapy with a uricosuric agent can be considered. However, uricosurics cannot be used in patients with a history of nephrolithiasis or with uricosuria levels higher than 700–800 mg/24 h. Finally, recombinant uricase such as pegloticase can be used in patients with refractory gout for a maximum of 6 months [102,114].

As previously mentioned, in the modern era, a holistic view of CV risk appears mandatory. Few medications used to treat other CV risk factors have been shown to collaterally reduce SUA levels. Two examples are the insulin-sensitizing troglitazone, used to treat patients with type 2 diabetes, and the fibrate fenofibrate, mainly used in the treatment of hypertriglyceridemia [115,116]. Knowing the UA-lowering properties of medications commonly used to treat other CV risk factors could help avoiding polytherapy and possible drug interactions, in particular in the elderly.

## 4. Conclusions

Almost one in three adults suffers from hypertension, with an increasing burden of disease worldwide. The most challenging consequence of hypertension is the so-called hypertension-mediated organ damage. It remains partially unexplained why some patients develop hypertension-mediated organ damage and others do not. Apart from efficient BP control strategies, other CV risk factors could play a synergistic effect with hypertension leading to organ damage and CVD development.

One of those CV risk factors is without a doubt UA. In fact, UA and hypertension are intimately associated. Several mechanisms for this association have been proposed, including RAAS regulation and systemic endoteliopathy. Anti-hypertensive drugs have been shown to influence SUA levels and, in turn, most hypouricemic agents have demonstrated an effect on BP values. In addition, pharmacological drug classes used to treat other CV risk factors, such as diabetes, have also shown to exert an effect on SUA levels.

A holistic approach to prevent and treat CV risk factors appears of critical importance. Large controlled studies on the effect of long-term anti-hyperuricemic agents on BP and CV risk reduction are warranted, with a specific focus in the highest risk population such as patients with gout and high SUA levels.

## Figures and Tables

**Table 1:** Studies on the prognostic role of UA in hypertension.

Study	*n*	Study Population	F-U	Results
Kahn et al., 1972 [4]	3829	Normotensive Israeli men aged ≥ 40 y at the enrollment	5 y	SUA was significantly associated with the incidence of hypertension
Selby et al., 1990 [6]	1900	950 Hypertensive and 950 Normotensive American Multiethnic cohort	6 y	SUA was an independent risk factor for development of hypertension
Hunt et al., 1991 [7]	1482	American adults belonging to 98 multigenerational pedigrees associated with the occurrence of coronary death, stroke death, or hypertension	7 y	SUA was associated with an increased risk of hypertension
Jossa et al., 1994 [8]	619	Normotensive Italian men enrolled in the Olivetti Heart Study	12 y	There was an independent positive association between serum uric acid levels and development of hypertension
Dyer et al., 1999 [9]	4195	American black and white adults aged 18–30 y at the enrollment in the CARDIA study	10 y	SUA was a predictor of 10-year incidence of hypertension
Taniguchi et al., 2001 [10]	6356	Japanese men aged 35–60 y, without hypertension and diabetes at the enrollment	10 y	SUA was associated with an increased risk for hypertension after adjustment for known risk factors
Nakanishi et al., 2003 [11]	2310	Japanese male office workers aged 35–59 y who did not have hypertension, impaired fasting glucose, Type II diabetes, or past history of cardiovascular disease at study entry	6 y	After controlling for potential predictors of hypertension and diabetes, the relative risk for hypertension compared with quintiles of SUA was progressively increased
Nagahama et al., 2004 [12]	4489	Japanese adults who did not have hypertension and were not currently using antihypertensive medication	3 y	Hyperuricemia predicted development of hypertension after multivariate analysis
Sundström et al., 2005 [13]	3329	Framingham Study participants (mean age 48.7 y; 55.6% women) free of hypertension, myocardial infarction, heart failure, renal failure, or gout	4 y	Age- and sex-adjusted rates of hypertension incidence increased progressively from 9.8% for the lowest quartile to 15.6% for the top quartile of SUA
Perlstein et al., 2006 [14]	2062	Healthy adult men	22 y	SUA independently predicted the development of hypertension in age-adjusted and multivariable models.
Mellen et al., 2006 [15]	9104	American black and white adults aged 45 to 64 y and without hypertension at baseline	9 y	Higher serum uric acid was associated with greater risk of hypertension in the overall cohort after multivariate adjustment
Shanker et al., 2006 [16]	2520	Adults from a population-based cohort study based in Beaver Dam city and township, Wisconsin, US	10 y	The relative risk of incident hypertension increased in a dose-dependent manner with increasing uric acid quartiles.
Krishnan et al., 2007 [17]	3073	Normotensive men with baseline hyperuricemia (serum uric acid >7.0 mg/dL) but without diabetes/glucose intolerance or metabolic syndrome	6 y	Men with hyperuricemia had an 80% excess risk for incident hypertension (hazard ratio: 1.81; 95% CI: 1.59 to 2.07)
Forman et al., 2009 [102]	1496	Women aged 32 to 52 y without hypertension at baseline	8 y	SUA was independently associated with incident hypertension
Kuwabara et al., 2018 [19]	3584	Prehypertensive Japanese adults	5 y	The cumulative incidence of hypertension in subjects with prehypertension and hyperuricemia was significantly higher than those without hyperuricemia
Cicero et al., 2019 [20]	376	Adults from Brisighella Heart Study cohort, Brisighella, Italy	10 y	The contemporary presence of suboptimal LDL-C and SUA values is associated with an increased risk to develop hypertension

List of abbreviations: CARDIA, Coronary Artery Risk Development in (Young) Adults Study; F-U, follow-up; LDL-C, low-density lipoprotein cholesterol; SUA, serum uric acid; y, years.

**Table 2:** Studies on the effects of UA-lowering drugs on BP.

Study	*n*	Study Population	Drug	F-U	Results
Kostka-Jeziorny et al., 2011 [46]	66	Adults with mild and moderate arterial hypertension	Allopurinol 150 mg/day	8 w	No significant changes in BP were observed after allopurinol treatment in either of the subgroups receiving ACE-I or thiazide-based antihypertensive therapy
Segal et al., 2015 [45]	110	African American adults with stage 1 hypertension without clinically significant renal disease who were receiving the thiazide-type diuretic chlorthalidone	Allopurinol 300 mg/day	8 w	The addition of allopurinol tended to improve BP, but the difference from the group receiving chlorthalidone alone was not statistically significant
Jalal et al., 2017 [44]	80	Adults with stage 3 CKD and asymptomatic hyperuricemia (≥7 mg/dL in men and ≥6 mg/dL in women)	Allopurinol 300 mg/day	12 w	No differences in BP were observed between treatment and placebo groups
McMullan et al., 2017 [48]	149	Overweight or obese adults with SUA ≥ 5.0 mg/dL	Probenecid 500 mg/day Allopurinol 300 mg/day	8 w	Uric acid lowering had no effect on kidney-specific or systemic RAS activity or on mean SBP
Borgi et al., 2017 [90]	149	Non-hypertensive, overweight, or obese individuals with higher serum uric acid	Probenecid 500/1000 mg/day Allopurinol 300–600 mg/day	8 w	Uric acid lowering did not impact endothelial function, a potential mechanism for development of hypertension
Kimura et al., 2018 [49]	441	Adults with UA > 7.0–10.0 mg/dL, stage 3 CKD, and no history of gout	Febuxostat 40 mg/day	27 m	The differences in mean estimates of DBP and SBP between groups were no significant, even if a trend towards an increased reduction in DBP was observed in the febuxostat group
Feig et al., 2008 [50]	30	Adolescents with newly diagnosed never-treated stage 1 essential hypertension and serum uric acid levels > or =6 mg/dL.	Allopurinol 400 mg/day	4 w	20/30 participants achieved normal BP while taking allopurinol vs. 1 participant while taking placebo (*p* < 0.001)
Kanbay et al., 2011 [57]	97	Adults with asymptomatic hyperuricemia and no history of gout	Allopurinol 300 mg/day	16 w	Allopurinol treatment resulted in a decrease in SBP
Soletsky et al., 2012 [55]	56	Children with prehypertension, hyperuricemia, and obesity	Allopurinol 400 mg/day, Probenecid 1000 mg/day	8 w	Subjects treated with urate lowering therapy experienced a highly significant reduction in BP
Kim et al., 2014 [53]	179	Men with gout and serum urate concentrations ≥ 8.0 mg/dL at screening	Febuxostat 40/80/120 mg/day, Allopurinol 300 mg/day	4 w	DBP decreased significantly in the allopurinol group. SBP decreased significantly in the allopurinol and febuxostat 120 mg/d group. However, adjustment for multiple variables affected the significance of these findings
Higgins et al., 2014 [56]	80	Adults with recent ischemic stroke or transient ischemic attack were eligible	Allopurinol 300 mg/day	12 m	Allopurinol lowered central BP at 1 year compared with placebo
Assadi, 2014 [51]	44	Adolescents newly diagnosed as essential hypertension, previously untreated.	Allopurinol 5 mg/kg/day (max 300 mg/day)	8 w	Adolescents randomized to enalapril plus allopurinol in combination showed greater BP reduction and percent of treatment group achieving target BP level compared to enalapril alone group
Tani et al., 2015 [59]	60	Hypertensive hyperuricemic patients	Febuxostat 40/80/120 mg/day	6 m	In the febuxostat group, the plasma renin activity, plasma aldosterone concentration, and SUA level significantly decreased
Madero et al., 2015 [52]	72	Prehypertensive and overweight subjects	Allopurinol 300 mg/day	4 w	Compared to placebo subjects, the allopurinol group had a lower clinic SBP, and this was significant within- and between-group comparisons
Gunawardhana et al., 2017 [54]	121	Adults with hyperuricemia without gout and stage I hypertension	Febuxostat 80 mg/day	6 w	There was a small, statistically significant difference between placebo and febuxostat in change from baseline SBP in the subgroup with normal renal function (mean difference −6.7; 95% CI, −13.3 to 0.0; *p* = 0.049)
Johnson et al., 2019 [58]	212	Adults with chronic refractory gout	Pegloticase 8 mg q2w and q4w	6 m	MAP was decreased by 5.0 mmHg from baseline to 6 months in the q2w responders

In the first section are reported the studies that did not show any UA-lowering drugs effect on BP. In the second section, the studies that showed an effect on BP of UA-lowering drugs are reported. Abbreviations: ACE-I, angiotensinogen converting enzyme Inhibitors; BP, blood pressure; CI, confidence intervals; CKD, chronic kidney disease; DBP, diastolic blood pressure; F-U, follow-up; MAP, mean arterial pressure; SBP, systolic blood pressure; SUA, serum uric acid; w, week; y, years.

## Data Availability

Not applicable.
